# Personal

**Published:** 1891-07

**Authors:** 


### Personal.

Dr. W. M. Ward, of North Collins, was elected president of the Lake Erie Medical Association at its annual meeting, held at Dayton, in April. The next meeting (quarterly) will be held in Angola, July 10 and 11, 1891.

---

Dr. and Mrs. Herman Mynter, of Buffalo, sailed on Wednesday, June 24th, for a trip through England, Denmark, and the continent, to return in the early autumn, in season for Dr. Mynter to resume his surgical teaching in the Niagara University Medical College.

---

Surgeon-General John B. Hamilton, U. S. Marine Hospital Service, has accepted the position of Professor of Surgery in the Rush Medical College, Chicago, Ill., recently declined by Professor Park, of this city ; so that, in the future, surgery will be taught in that institution by two professors of equal rank.

---

Dr. Carl H. Von Klein, formerly of Dayton, O., has removed to Cleveland, and established himself at 122 Euclid avenue. Dr. Von Klein has been for many years noted as a specialist in diseases of the respiratory tract, and will continue in the same line of practice in his newly-chosen home. We bespeak for him the continued confidence of the profession that his skill and experience merits.

---

Dr. Theo. G. Lewis having been given the management of the manufacturing department of the Buffalo Dental Supply and Manufacturing Co., has been succeeded in his practice by Dr. Frank W. Low, who for four years has been Dr. Lewis's assistant. Dr. Lewis commends Dr. Low as entirely trustworthy and competent, and trusts that former patients will continue their patronage at the old office, No. 15 Court street.

---

Surgeon-General Walter Wyman.—Dr. Jno. B. Hamilton having resigned the position of Surgeon-General in the Marine Hospital Service to accept the chair of Surgery in the Rush Medical College, of Chicago, Dr. Walter Wyman, who for a long time had been the assistant of Dr. Hamilton, was, upon the recommendation of the latter, appointed by the President to fill the vacancy.—*Memphis Medical Monthly.*

